# Crystal structure of (*E*)-4-meth­oxy-2-{[(5-methyl­pyridin-2-yl)imino]­meth­yl}phenol

**DOI:** 10.1107/S2056989015018113

**Published:** 2015-10-03

**Authors:** Farook Adam, Md Azharul Arafath, Rosenani Anwaeul Haque, Mohd Rizal Razali

**Affiliations:** a School of Chemical Sciences, 11800, USM Pulau Pinang, Malaysia

**Keywords:** crystal structure, Schiff base, *N*-heterocycle, intra­molecular hydrogen bond

## Abstract

The mol­ecule of the title Schiff base compound, C_14_H_14_N_2_O_2_, displays an *E* conformation with respect the imine C=N double bond. The mol­ecule is approximately planar, with the dihedral angle formed by the planes of the pyridine and benzene rings being 5.72 (6)°. There is an intra­molecular hydrogen bond involving the phenolic H and imine N atoms.

## Related literature   

For the structure of related *N*-heterocyclic Schiff base compounds, see: Sahebalzamani *et al.* (2011 ▸); Rawat & Singh (2015 ▸); Thakar *et al.* (2015 ▸); Salam *et al.* (2011 ▸).
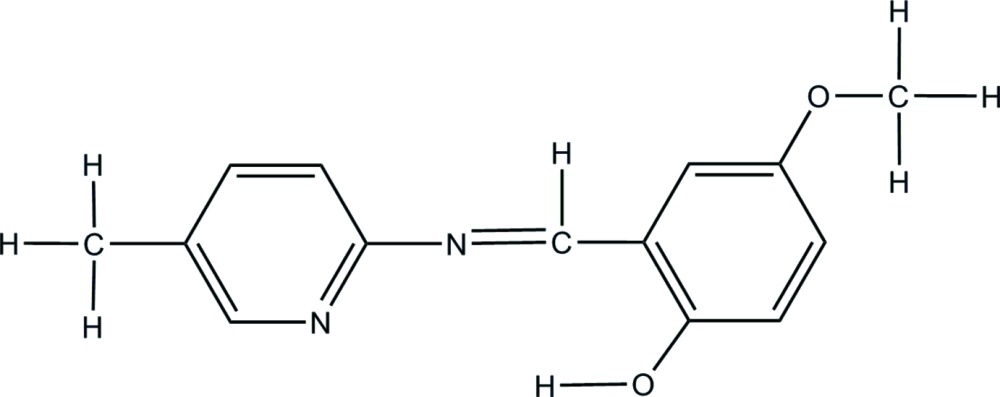


## Experimental   

### Crystal data  

C_14_H_14_N_2_O_2_
*M* *_r_* = 242.27
Monoclinic 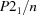

*a* = 12.7082 (12) Å
*b* = 4.7446 (4) Å
*c* = 21.124 (2) Å
β = 105.525 (2)°
*V* = 1227.21 (19) Å^3^
*Z* = 4
Mo *K*α radiation
μ = 0.09 mm^−1^
*T* = 294 K
0.41 × 0.35 × 0.12 mm

### Data collection  

Bruker APEXII CCD diffractometer
Absorption correction: multi-scan (SABABS; Bruker, 2014 ▸) *T*_min_ = 0.030, *T*_max_ = 0.262
10950 measured reflections
2848 independent reflections
1910 reflections with *I* > 2σ(*I*)
*R*_int_ = 0.028

### Refinement  

*R*[*F* ^2^ > 2σ(*F* ^2^)] = 0.044
*wR*(*F* ^2^) = 0.151
*S* = 1.07
2848 reflections
170 parameters
H atoms treated by a mixture of independent and constrained refinement
Δρ_max_ = 0.17 e Å^−3^
Δρ_min_ = −0.18 e Å^−3^

### 

Data collection: *APEX2* (Bruker, 2014 ▸); cell refinement: *SAINT* (Bruker, 2014 ▸); data reduction: *SAINT*; program(s) used to solve structure: *SHELXS97* (Sheldrick, 2008 ▸); program(s) used to refine structure: *SHELXL2013* (Sheldrick, 2015 ▸); molecular graphics: *SHELXTL* (Sheldrick, 2008 ▸); software used to prepare material for publication: *SHELXTL*.

## Supplementary Material

Crystal structure: contains datablock(s) I. DOI: 10.1107/S2056989015018113/rz5166sup1.cif

Structure factors: contains datablock(s) I. DOI: 10.1107/S2056989015018113/rz5166Isup2.hkl

Supporting information file. DOI: 10.1107/S2056989015018113/rz5166Isup3.cml

**Fig. 1.**
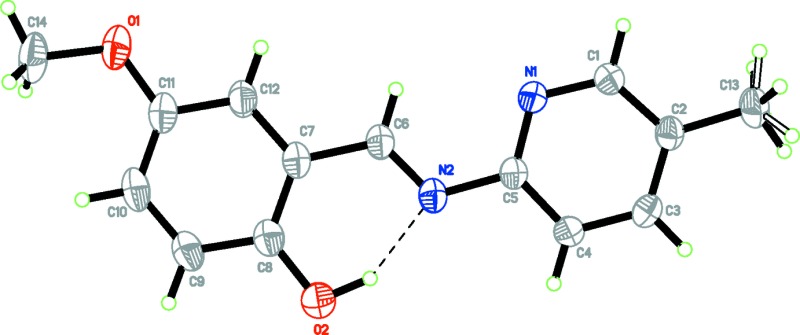
The molecular structure of the title compound with displacement ellipsoids drawn at the 30% probability level. The dashed line indicates an intramolecular hydrogen bond.

**Fig. 2.**
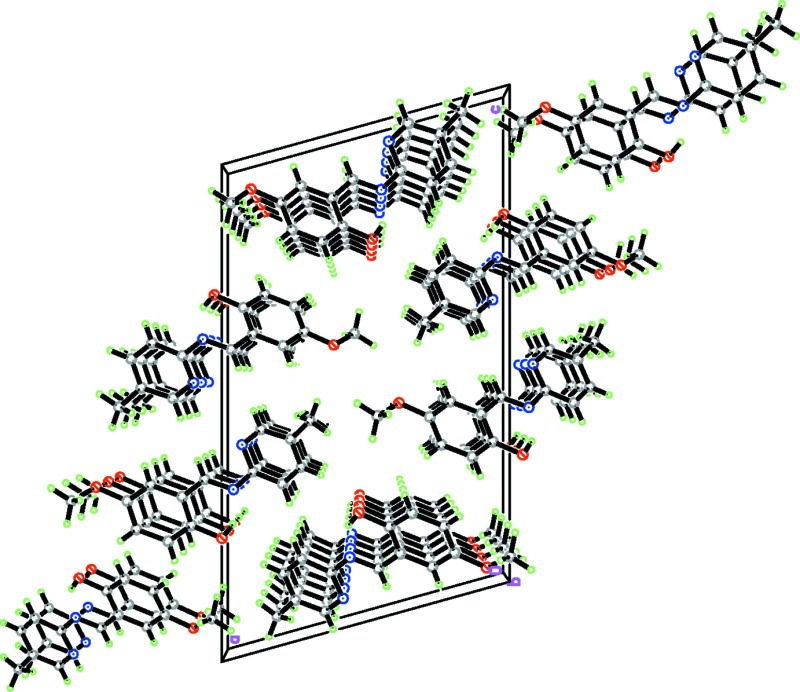
Packing of the title compound compound viewed down the b axis.

. DOI: 10.1107/S2056989015018113/rz5166fig1.tif
The mol­ecular structure of the title compound with displacement ellipsoids drawn at the 30% probability level. The dashed line indicates an intra­molecular hydrogen bond.

b . DOI: 10.1107/S2056989015018113/rz5166fig2.tif
Packing of the title compound compound viewed down the *b* axis.

**Fig. 3.**
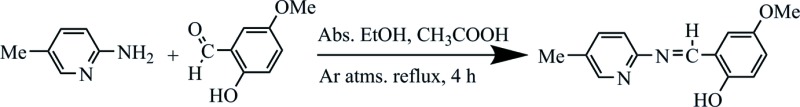
Synthesis of the title compound.

. DOI: 10.1107/S2056989015018113/rz5166fig3.tif
Synthesis of the title compound.

CCDC reference: 1048553

Additional supporting information:  crystallographic information; 3D view; checkCIF report

## Figures and Tables

**Table 1:** Hydrogen-bond geometry (, )

*D*H*A*	*D*H	H*A*	*D* *A*	*D*H*A*
O2H1*O*2N2	0.95(2)	1.76(3)	2.6276(19)	150(2)

## supplementary crystallographic information

### S1. Comment

The molecule of the title compound (Fig. 1) has an *E* configuration about the imine C═N double bond, as indicated by the value of 179.11(12° of the C5-N2-C6-C7 torsion angle. The molecule is is almost planar, with a dihedral angle between the pyridine and benzene rings of 5.72 (6)°. The molecular conformation is stabilized by an intramolecular O—H···N hydrogen bond (Table 1) occurring between the phenolic hydrogen and imine nitrogen atoms. In the crystal, packing is stabilized only by van der Waals interactions.

### S2. Experimental

2-Hydroxy-5-methoxybenzaldehyde (5 mmol, 0.761 g) and 2-amino-5-methylpyridin (5 mmol, 0.541 g) were dissolved in ethanol in separate beakers, then the amine solution was added drop wise with stirring to the aldehyde in the round bottomed flask. Acetic acid was added and the solution was refluxed for 4 h under Ar atmosphere (Fig. 3). The solid product obtained was dried under reduced pressure overnight, then recrystallized with dichloromethane, diethyl ether and excess n-hexane, filtered, washed again with diethyl ether/n-hexane (1:3 *v*/*v*) and dried out over 24 h under reduced pressure in a desiccator. Purple single crystals of the title compound were grown on evaporation of an ethanol solution. M. p.: 380-381 K. Yield: 85%. Anal. calc. for C_14_H_14_N_2_O_2_ (FW: 242.28 g/mol): C, 69.43; H, 5.77; N, 11.55%. Found: C, 69.93; H, 5.69; N, 11.54%. IR (KBr pellets µ_max_/cm^-1^): 3427 ν(N—H), 2952 and 1384 ν(CH_3_), 1618 ν(C═N), 1497 ν(C═C, ar.), 1210 ν(C—O), 1035 ν(C—N).

### S3. Refinement

The phenolic hydrogen atom was located in a difference Fourier map and refined freely. All other H atoms were calculated geometrically and refined using a riding model, with C—H = 0.93 Å, and with *U*_iso_(H) = 1.2 *U*_eq_(C) or 1.5 *U*_eq_(C) for methyl H atoms. A rotating model was used for all methyl group. The H atoms of the methyl carbon attached to the pyridine ring are disordered over two sets of sites with an occupancy ratio of 0.61 (2):0.39 (2).

### Crystal data

**Table:**

C_14_H_14_N_2_O_2_	*F*(000) = 512
*M**_r_* = 242.27	*D*_x_ = 1.311 Mg m^−^^3^
Monoclinic, *P*2_1_/*n*	Melting point: 380 K
*a* = 12.7082 (12) Å	Mo *K*α radiation, λ = 0.71073 Å
*b* = 4.7446 (4) Å	θ = 1.7–27.6°
*c* = 21.124 (2) Å	µ = 0.09 mm^−^^1^
β = 105.525 (2)°	*T* = 294 K
*V* = 1227.21 (19) Å^3^	Block, purple
*Z* = 4	0.41 × 0.35 × 0.12 mm

### Data collection

**Table:**

Bruker APEXII CCD diffractometer	1910 reflections with *I* > 2σ(*I*)
φ and ω scans	*R*_int_ = 0.028
Absorption correction: multi-scan (SABABS; Bruker, 2014)	θ_max_ = 27.6°, θ_min_ = 1.7°
*T*_min_ = 0.030, *T*_max_ = 0.262	*h* = −16→14
10950 measured reflections	*k* = −6→6
2848 independent reflections	*l* = −27→27

### Refinement

**Table:**

Refinement on *F*^2^	0 restraints
Least-squares matrix: full	Hydrogen site location: mixed
*R*[*F*^2^ > 2σ(*F*^2^)] = 0.044	H atoms treated by a mixture of independent and constrained refinement
*wR*(*F*^2^) = 0.151	*w* = 1/[σ^2^(*F*_o_^2^) + (0.0755*P*)^2^ + 0.121*P*] where *P* = (*F*_o_^2^ + 2*F*_c_^2^)/3
*S* = 1.07	(Δ/σ)_max_ < 0.001
2848 reflections	Δρ_max_ = 0.17 e Å^−^^3^
170 parameters	Δρ_min_ = −0.18 e Å^−^^3^

### Special details

**Table:**

Geometry. All e.s.d.'s (except the e.s.d. in the dihedral angle between two l.s. planes) are estimated using the full covariance matrix. The cell e.s.d.'s are taken into account individually in the estimation of e.s.d.'s in distances, angles and torsion angles; correlations between e.s.d.'s in cell parameters are only used when they are defined by crystal symmetry. An approximate (isotropic) treatment of cell e.s.d.'s is used for estimating e.s.d.'s involving l.s. planes.

### Fractional atomic coordinates and isotropic or equivalent isotropic displacement parameters (Å^2^)

**Table:**

	*x*	*y*	*z*	*U*_iso_*/*U*_eq_	Occ. (<1)
O1	0.11027 (10)	0.5504 (3)	0.07471 (7)	0.0866 (5)	
O2	0.52254 (10)	0.4026 (3)	0.24106 (6)	0.0762 (4)	
N1	0.57565 (9)	−0.2914 (3)	0.07413 (6)	0.0527 (3)	
N2	0.55236 (9)	0.0280 (3)	0.15589 (6)	0.0495 (3)	
C1	0.63754 (12)	−0.4827 (3)	0.05470 (8)	0.0543 (4)	
H1A	0.6101	−0.5632	0.0134	0.065*	
C2	0.73934 (11)	−0.5698 (3)	0.09138 (7)	0.0476 (4)	
C3	0.77704 (12)	−0.4503 (4)	0.15259 (8)	0.0558 (4)	
H3A	0.8446	−0.5033	0.1797	0.067*	
C4	0.71504 (12)	−0.2527 (4)	0.17389 (7)	0.0548 (4)	
H4A	0.7403	−0.1707	0.2152	0.066*	
C5	0.61512 (10)	−0.1780 (3)	0.13323 (7)	0.0445 (3)	
C6	0.45685 (12)	0.0882 (3)	0.12034 (8)	0.0521 (4)	
H6A	0.4309	−0.0023	0.0801	0.063*	
C7	0.38731 (11)	0.2931 (3)	0.14055 (7)	0.0487 (4)	
C8	0.42234 (12)	0.4410 (3)	0.19963 (7)	0.0536 (4)	
C9	0.35180 (14)	0.6348 (4)	0.21589 (8)	0.0630 (5)	
H9A	0.3750	0.7380	0.2545	0.076*	
C10	0.24841 (14)	0.6764 (3)	0.17591 (9)	0.0621 (4)	
H10A	0.2021	0.8053	0.1880	0.074*	
C11	0.21273 (13)	0.5282 (4)	0.11786 (9)	0.0593 (4)	
C12	0.28284 (12)	0.3399 (4)	0.10047 (8)	0.0583 (4)	
H12A	0.2596	0.2422	0.0610	0.070*	
C13	0.80422 (14)	−0.7829 (4)	0.06515 (9)	0.0651 (5)	
H13A	0.8653	−0.8443	0.0999	0.098*	0.61 (2)
H13B	0.8302	−0.6989	0.0308	0.098*	0.61 (2)
H13C	0.7587	−0.9416	0.0479	0.098*	0.61 (2)
H13D	0.8763	−0.7110	0.0692	0.098*	0.39 (2)
H13E	0.7689	−0.8191	0.0197	0.098*	0.39 (2)
H13F	0.8090	−0.9547	0.0897	0.098*	0.39 (2)
C14	0.03899 (15)	0.7589 (5)	0.08715 (13)	0.0898 (7)	
H14A	−0.0274	0.7588	0.0523	0.135*	
H14B	0.0732	0.9404	0.0895	0.135*	
H14C	0.0227	0.7192	0.1281	0.135*	
H1O2	0.5562 (19)	0.261 (5)	0.2213 (13)	0.120 (9)*	

### Atomic displacement parameters (Å^2^)

**Table:**

	*U*^11^	*U*^22^	*U*^33^	*U*^12^	*U*^13^	*U*^23^
O1	0.0597 (7)	0.0913 (10)	0.1072 (10)	0.0322 (7)	0.0198 (7)	−0.0079 (8)
O2	0.0739 (8)	0.0858 (10)	0.0632 (7)	0.0210 (7)	0.0084 (6)	−0.0106 (7)
N1	0.0450 (6)	0.0544 (7)	0.0554 (7)	0.0084 (6)	0.0076 (5)	−0.0070 (6)
N2	0.0510 (7)	0.0454 (7)	0.0547 (7)	0.0090 (5)	0.0186 (5)	0.0028 (6)
C1	0.0491 (8)	0.0565 (9)	0.0558 (8)	0.0042 (7)	0.0113 (6)	−0.0122 (7)
C2	0.0453 (7)	0.0406 (7)	0.0595 (8)	0.0022 (6)	0.0182 (6)	0.0003 (7)
C3	0.0449 (7)	0.0614 (10)	0.0567 (8)	0.0126 (7)	0.0057 (6)	0.0021 (8)
C4	0.0527 (8)	0.0620 (10)	0.0461 (7)	0.0114 (7)	0.0067 (6)	−0.0049 (7)
C5	0.0448 (7)	0.0409 (7)	0.0494 (7)	0.0047 (6)	0.0151 (6)	0.0012 (6)
C6	0.0520 (8)	0.0489 (8)	0.0578 (8)	0.0094 (7)	0.0187 (7)	0.0000 (7)
C7	0.0524 (8)	0.0419 (7)	0.0576 (8)	0.0073 (6)	0.0247 (7)	0.0058 (7)
C8	0.0619 (9)	0.0489 (8)	0.0553 (8)	0.0079 (7)	0.0248 (7)	0.0076 (7)
C9	0.0803 (11)	0.0557 (9)	0.0618 (9)	0.0118 (9)	0.0342 (9)	−0.0001 (8)
C10	0.0730 (10)	0.0500 (9)	0.0774 (11)	0.0155 (8)	0.0448 (9)	0.0074 (8)
C11	0.0554 (9)	0.0545 (9)	0.0749 (10)	0.0134 (7)	0.0293 (8)	0.0078 (8)
C12	0.0557 (8)	0.0558 (9)	0.0663 (9)	0.0131 (8)	0.0213 (7)	−0.0019 (8)
C13	0.0633 (9)	0.0536 (9)	0.0842 (11)	0.0102 (8)	0.0297 (9)	−0.0057 (9)
C14	0.0614 (10)	0.0771 (13)	0.1362 (19)	0.0267 (10)	0.0357 (11)	0.0035 (13)

### Geometric parameters (Å, º)

**Table:**

O1—C11	1.379 (2)	C7—C12	1.388 (2)
O1—C14	1.413 (2)	C7—C8	1.397 (2)
O2—C8	1.3505 (19)	C8—C9	1.390 (2)
O2—H1O2	0.95 (3)	C9—C10	1.373 (2)
N1—C5	1.3282 (18)	C9—H9A	0.9300
N1—C1	1.3356 (18)	C10—C11	1.381 (2)
N2—C6	1.2771 (18)	C10—H10A	0.9300
N2—C5	1.4231 (17)	C11—C12	1.379 (2)
C1—C2	1.381 (2)	C12—H12A	0.9300
C1—H1A	0.9300	C13—H13A	0.9600
C2—C3	1.375 (2)	C13—H13B	0.9600
C2—C13	1.501 (2)	C13—H13C	0.9600
C3—C4	1.376 (2)	C13—H13D	0.9600
C3—H3A	0.9300	C13—H13E	0.9600
C4—C5	1.3754 (19)	C13—H13F	0.9600
C4—H4A	0.9300	C14—H14A	0.9600
C6—C7	1.4526 (19)	C14—H14B	0.9600
C6—H6A	0.9300	C14—H14C	0.9600
			
C11—O1—C14	118.00 (16)	C10—C9—H9A	119.4
C8—O2—H1O2	105.5 (15)	C8—C9—H9A	119.4
C5—N1—C1	117.31 (12)	C9—C10—C11	120.38 (14)
C6—N2—C5	119.06 (13)	C9—C10—H10A	119.8
N1—C1—C2	124.82 (14)	C11—C10—H10A	119.8
N1—C1—H1A	117.6	C12—C11—O1	115.88 (16)
C2—C1—H1A	117.6	C12—C11—C10	118.98 (16)
C3—C2—C1	116.25 (13)	O1—C11—C10	125.14 (14)
C3—C2—C13	122.49 (14)	C11—C12—C7	121.46 (16)
C1—C2—C13	121.26 (14)	C11—C12—H12A	119.3
C2—C3—C4	120.18 (13)	C7—C12—H12A	119.3
C2—C3—H3A	119.9	C2—C13—H13A	109.5
C4—C3—H3A	119.9	C2—C13—H13B	109.5
C5—C4—C3	119.02 (14)	H13A—C13—H13B	109.5
C5—C4—H4A	120.5	C2—C13—H13C	109.5
C3—C4—H4A	120.5	H13A—C13—H13C	109.5
N1—C5—C4	122.41 (13)	H13B—C13—H13C	109.5
N1—C5—N2	119.32 (12)	C2—C13—H13D	109.5
C4—C5—N2	118.27 (13)	C2—C13—H13E	109.5
N2—C6—C7	122.28 (14)	H13D—C13—H13E	109.5
N2—C6—H6A	118.9	C2—C13—H13F	109.5
C7—C6—H6A	118.9	H13D—C13—H13F	109.5
C12—C7—C8	119.25 (13)	H13E—C13—H13F	109.5
C12—C7—C6	119.06 (14)	O1—C14—H14A	109.5
C8—C7—C6	121.69 (14)	O1—C14—H14B	109.5
O2—C8—C9	119.18 (15)	H14A—C14—H14B	109.5
O2—C8—C7	122.08 (13)	O1—C14—H14C	109.5
C9—C8—C7	118.74 (15)	H14A—C14—H14C	109.5
C10—C9—C8	121.17 (16)	H14B—C14—H14C	109.5
			
C5—N1—C1—C2	−0.3 (2)	C12—C7—C8—O2	179.28 (14)
N1—C1—C2—C3	1.0 (2)	C6—C7—C8—O2	0.2 (2)
N1—C1—C2—C13	−178.93 (14)	C12—C7—C8—C9	−1.1 (2)
C1—C2—C3—C4	−0.9 (2)	C6—C7—C8—C9	179.78 (13)
C13—C2—C3—C4	178.97 (14)	O2—C8—C9—C10	−178.66 (14)
C2—C3—C4—C5	0.3 (2)	C7—C8—C9—C10	1.7 (2)
C1—N1—C5—C4	−0.5 (2)	C8—C9—C10—C11	−0.9 (2)
C1—N1—C5—N2	179.64 (12)	C14—O1—C11—C12	−174.24 (16)
C3—C4—C5—N1	0.5 (2)	C14—O1—C11—C10	6.3 (3)
C3—C4—C5—N2	−179.64 (13)	C9—C10—C11—C12	−0.6 (2)
C6—N2—C5—N1	3.8 (2)	C9—C10—C11—O1	178.92 (15)
C6—N2—C5—C4	−176.11 (13)	O1—C11—C12—C7	−178.37 (14)
C5—N2—C6—C7	179.11 (12)	C10—C11—C12—C7	1.2 (3)
N2—C6—C7—C12	−177.58 (14)	C8—C7—C12—C11	−0.3 (2)
N2—C6—C7—C8	1.5 (2)	C6—C7—C12—C11	178.81 (14)

### Hydrogen-bond geometry (Å, º)

**Table:**

*D*—H···*A*	*D*—H	H···*A*	*D*···*A*	*D*—H···*A*
O2—H1*O*2···N2	0.95 (2)	1.76 (3)	2.6276 (19)	150 (2)
