# Supplementary figures and images for: Crystal structure of (E)-4-meth­oxy-2-{[(5-methyl­pyridin-2-yl)imino]­meth­yl}phenol
Source: Acta Crystallogr E Crystallogr Commun. 2015 Oct 3;71(Pt 11):o819. doi: 10.1107/S2056989015018113 (PMC4645057; doi:10.1107/S2056989015018113)

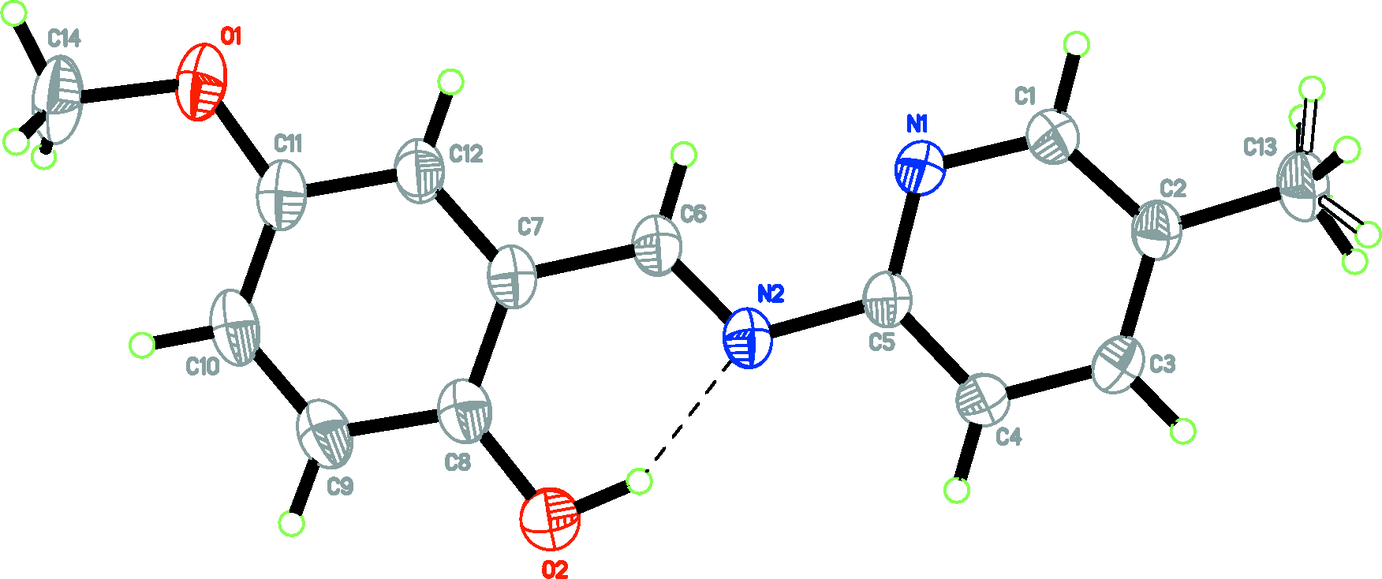

Supplement: Supplementary file 4 [file e-71-0o819-fig1.tif]

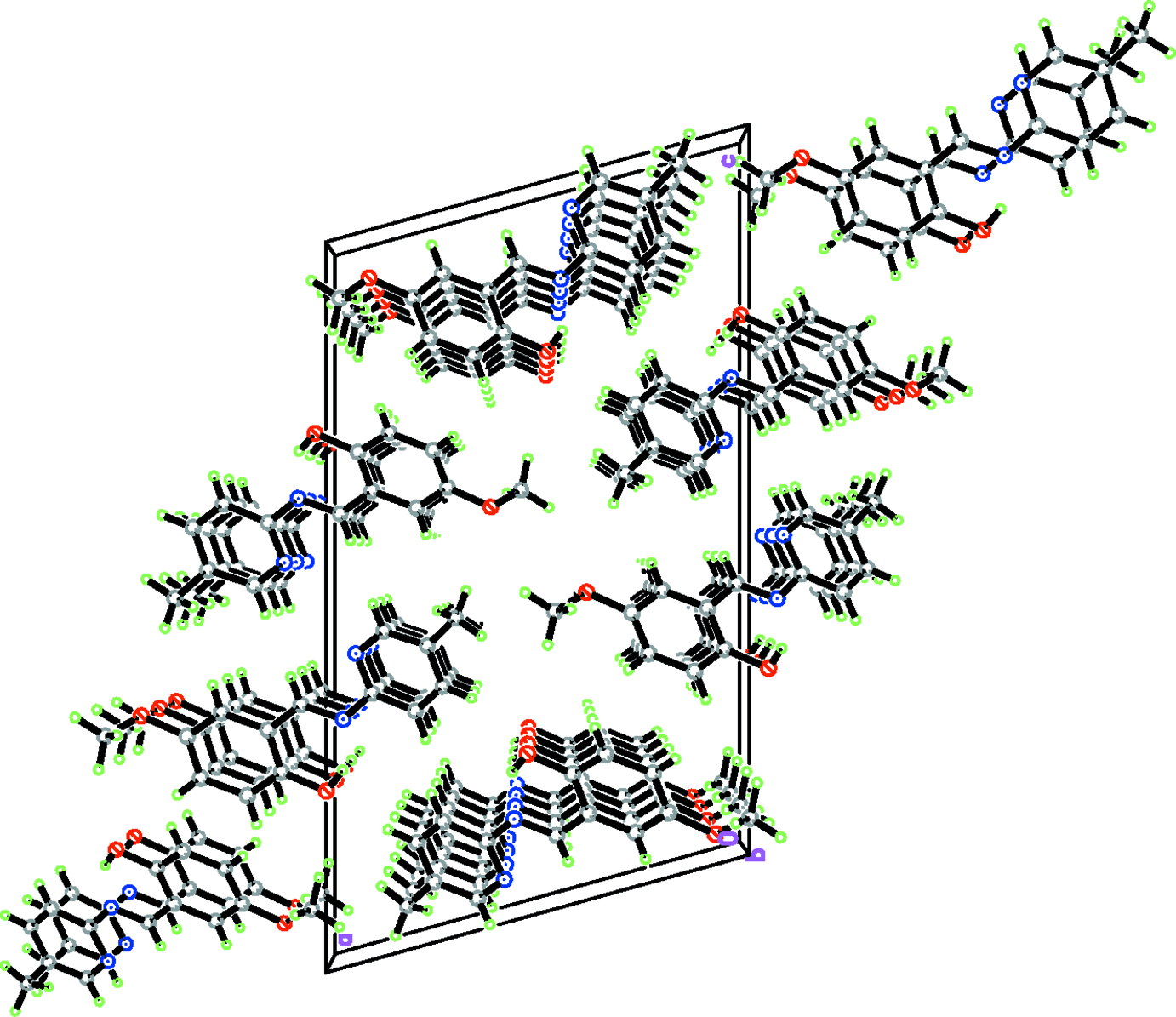

Supplement: Supplementary file 5 [file e-71-0o819-fig2.tif]

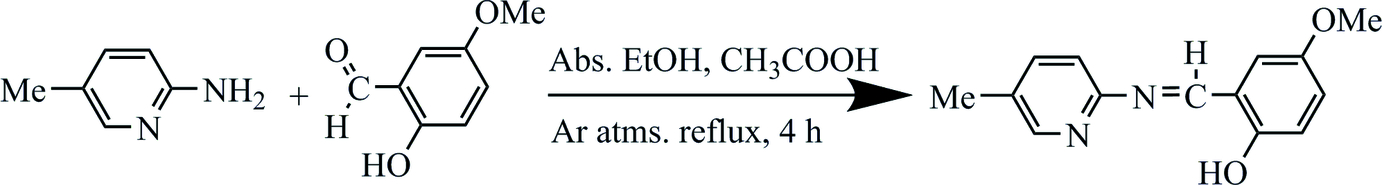

Supplement: Supplementary file 6 [file e-71-0o819-fig3.tif]
